# Proteome Mapping of Cervical Mucus and Its Potential as a Source of Biomarkers in Female Tract Disorders

**DOI:** 10.3390/ijms24021038

**Published:** 2023-01-05

**Authors:** Tomáš Oždian, Jan Vodička, Jiří Dostál, Dušan Holub, Jana Václavková, Michal Ješeta, Barbora Hamerníková, Pavla Kouřilová, Ondřej Malchar, Vladimír Dvořák, Pavel Hejtmánek, Kateřina Sobková, Pavel Ventruba, Radovan Pilka, Petr Džubák, Marián Hajdúch

**Affiliations:** 1 Laboratory of Experimental Medicine, Institute of Molecular and Translational Medicine, Faculty of Medicine and Dentistry, Palacký University, Hněvotínská 5, 779 00 Olomouc, Czech Republic; 2 Department of Gynecology and Obstetrics, University Hospital in Olomouc, Faculty of Medicine and Dentistry, Palacký University in Olomouc, Hněvotínská 3, 779 00 Olomouc, Czech Republic; 3 Department of Gynecology and Obstetrics, University Hospital in Olomouc, I.P.Pavlova 6, 779 00 Olomouc, Czech Republic; 4 Department of Gynecology and Obstetrics, University Hospital Brno, Obilni trh 11, 625 00 Brno, Czech Republic; 5 Department of Gynecology and Obstetrics, Masaryk University Brno, Obilni trh 11, 625 00 Brno, Czech Republic; 6 Laboratory of Experimental Medicine, University Hospital in Olomouc, Hněvotínská 5, 779 00 Olomouc, Czech Republic

**Keywords:** proteomics, cervical mucus, gynecology, intrauterine insemination, in vitro fertilization

## Abstract

Cervical mucus (CM) is a viscous fluid that is produced by the cervical glands and functions as a uterine cervix plug. Its viscosity decreases during ovulation, providing a window for non-invasive sampling. This study focuses on proteomic characterization of CM to evaluate its potential as a non-invasively acquired source of biomarkers and in understanding of molecular (patho)physiology of the female genital tract. The first objective of this work was to optimize experimental workflow for CM processing and the second was to assess differences in the proteomic composition of CM during natural ovulatory cycles obtained from intrauterine insemination (IUI) cycles and in vitro fertilization (IVF) cycles with controlled ovarian hyperstimulation. Proteomic analysis of CM samples revealed 4370 proteins involved in processes including neutrophil degranulation, cellular stress responses, and hemostasis. Differential expression analysis revealed 199 proteins enriched in IUI samples and 422 enriched in IVF. The proteins enriched in IUI were involved in phosphatidic acid synthesis, responses to external stimulus, and neutrophil degranulation, while those enriched in IVF samples were linked to neutrophil degranulation, formation of a cornified envelope and hemostasis. Subsequent analyses clarified the protein composition of the CM and how it is altered by hormonal stimulation of the uterus.

## 1. Introduction

Cervical mucus is a gel containing a plethora of glycoproteins, lipids, nucleic acids, and metabolites. Its biological function depends heavily upon macromolecular composition. The mucus plays a vital role in protecting the vaginal epithelium during sexual intercourse and in the innate protection of the uterine cavity, thereby helping to ensure fertility [1], sperm capacitation, and restricting sperm passage within the female reproductive tract (FRT) to the periovulatory period. However, its functions are not yet fully understood. Moreover, the biological and chemical–physical properties of cervical mucus change during the menstrual cycle [1,2]: its content of glycoproteins (mucins) peaks near ovulation due to high secretion, but the abundance of other proteins is highest during the luteal phase [3]. During the first half of the cycle, the mucus is scant, thick and viscous, forming a barrier or cervical plug that limits the access of sperm to the upper genital tract. Then, immediately before ovulation, when estrogens are produced in increasing amounts, its viscosity decreases while its volume increases 10- to 20-fold, thereby maximizing its permeability to spermatozoa. The composition of cervical mucus changes immediately after ovulation when the corpus luteum of the ovary begins synthesizing progesterone. Progesterone reduces the quantity and composition of cervical mucus, making it thicker and stickier [4]. The biochemical processes underlying these changes are poorly understood, but it is known that changes in the number and types of polysaccharide chains (glycans) attached to proteins during post-translational modification can drastically increase the proteins’ molecular weights and thereby alter their solubility, solution viscosity, and biological function. 

The uterine cavity, which is directly connected to the cervix, is coated with endometrial tissue that undergoes physiological remodeling induced by ovarian hormones. The human uterine proteome during the menstrual cycle has been studied by analyzing both the endometrial epithelium [5] and the uterine fluid [6]. However, the variation of the proteome of the uterine fluid over the course of the cycle is unknown. In addition, because the uterine cavity releases substances into the uterine fluid that may influence the entire reproductive system, the cervical mucus’s proteome may reflect the functional status of the endometrial tissue [7] and potentially also the fallopian tubes and ovaries. Thus, cervical mucus analysis could provide valuable information to support diagnosis, prognosis, and therapy of female tract disorders. Early detection of markers in cervical fluid proximal to diseased tissues may replace of complement venous blood and thus increase sensitivity of detection and in turn the range of therapeutic options available for individuals affected by diseases, enable more effective early treatment, and facilitate the identification of factors involved in pathogenesis.

However, only a few studies have investigated the protein composition of cervical mucus. Andersch-Björkman et al. [8] reported comprehensive proteomic and glycomic LC-MS analyses of cervical mucus samples collected from 12 women at multiple stages of the menstrual cycle (before, during, and after ovulation), while Panicker et al. optimized a SELDI-TOF (surface-enhanced laser desorption/ionization with time-of-flight mass analyzer) MS method for cervical mucus analysis [9]. Additionally, Grande et al. confirmed that cervical mucus is a rich source of protein biomarkers, determined the protein composition of cervical mucus samples from five fertile women with term delivery during the year preceding the study, and characterized the changes in the cervical mucus proteome during the menstrual cycle [10]. In another study, high-resolution mass spectrometric analysis of cervical mucus from ten infertile women with ovarian endometriotic cysts revealed that this chronic disease is characterized by inflammatory protein expression [11]. Changes in expression levels of cytokines (IFN-gamma, CM-CSF, RANTES, and eotaxin) in cervical mucus were described by Otani et al. (2019) in a patient with cervical dysplasia and cervical cancer [12]. Finally, Rocconi et al. (2021) recently analyzed the cervical mucus proteome for the purposes of ovarian cancer screening [13], and Finan et al. (2012) identified 10 cervical mucus proteins that can be used to confidently diagnose endometrial cancer [14]. These findings clearly show that the cervical mucus proteome contains information that could be valuable in diagnosing diverse pathologies and abnormalities in humans.

Although several proteomic analyses of cervical mucus were reported previously, the studies usually suffer from a low number of enrolled patients, and several studies sampled mucus irrespectively of the menstrual cycle. The definition of the normal cervical mucus proteome is particularly complicated, as we know its composition is heavily dependent on the ovarian cycle and estrogen levels. In order to standardize the sampling of biological material, we performed non-invasive sampling of cervical mucus during the periovulation period in healthy women undergoing artificial insemination or in vitro fertilization (IVF) due to male factor infertility. Experiments have been conducted to determine whether removing cervical mucus during intra-uterine insemination (IUI) might increase pregnancy rates [15], and there is evidence that clomiphene citrate (a drug used for ovarian stimulation in IVF fertility treatment) can alter the quality of cervical mucus [16,17]. IUI is based on women’s natural cycles and thus represents the normal biology of cervical mucus, whereas IVF-stimulated cycles represent a cervical mucus “model system” in which hormonal medication is administered with a standardized timing that allows for the timing of ovulation to be predicted very accurately, making it possible to sample the cervical mucus directly at the time of ovum pick-up.

## 2. Results

### 2.1. Patient Characteristics

The clinical characteristics of the participants in the study are summarized in Table 1.

### 2.2. Quality Control of the Dataset

The LC-MS analyses of all samples examined in this study were performed in a single batch. The stability of the analyses was evaluated retrospectively using the doubly charged peptide YICDNQDTISSK as a reference analyte. This peptide had a retention time of 29.22 ± 0.32 min (1% RSD), with a FWHM of 0.13 ± 0.007 min (5.5% RSD), a maximal peak height of 6.19 × 10^8^ ± 4.11 × 10^7^ (6.6% RSD), and an average mass error of 0.57 ± 0.57 ppm. All these values are below the 15% RSD cutoff commonly applied in LC-MS analyses, and the peptide’s mass error was below the 3 ppm cut-off used internally in our facility.

### 2.3. Proteomic Characterization of the Cervical Mucus

**Figure 1:**
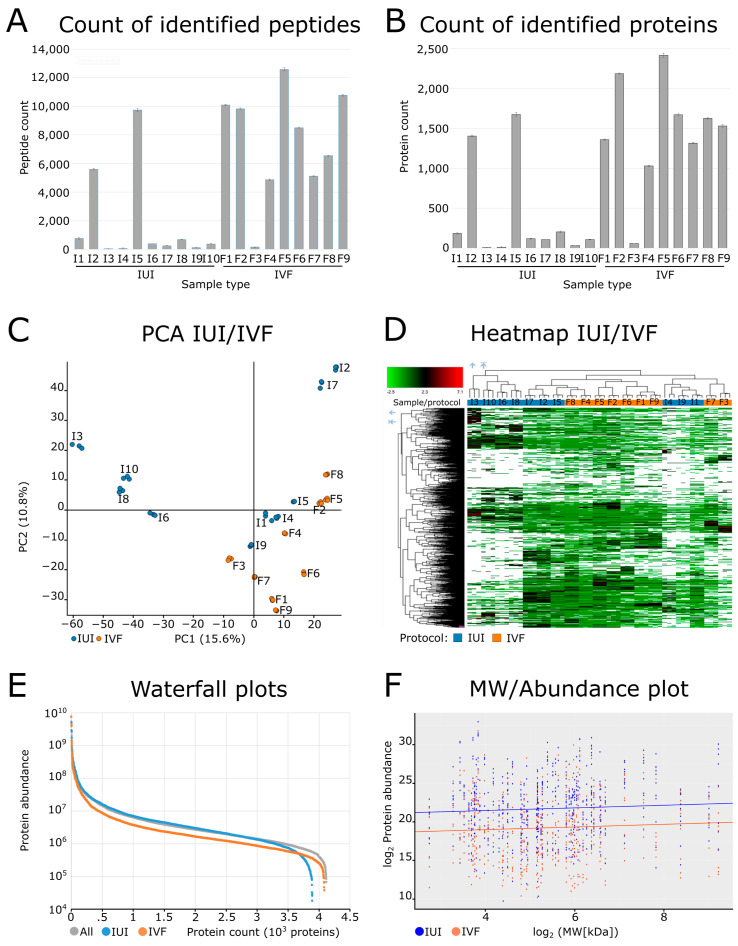
Proteomic characterization of the analyzed samples. Graph (**A**) shows the counts of peptides identified in each sample (one bar corresponds to the average of three technical replicates ± standard deviation). Graph (**B**) shows the counts of proteins in each sample (one bar corresponds to the average of three technical replicates ± standard deviation). The PCA plot (**C**) and heatmap (**D**) illustrate global differences among samples. The PCA plot is centered and scaled. The heatmap parameters were scaled before clustering and calculating squared Euclidean distances. The waterfall plot (**E**) shows the distribution of normalized protein abundances. The MW/abundance plot (**F**) shows the normalized protein abundances and molecular weights of the 63 proteins that were identified in all samples and replicates in IUI and IVF groups, respectively.

In the final search, a total of 4370 proteins (Table S1), 34,166 peptides, and 470,013 peptide spectrum matches were identified over all 19 samples collectively using ProteomeDiscoverer 2.5. The counts of identified peptides and proteins differed significantly between the IUI and IVF groups (Figure 1A,B). For the IUI group, the average number of proteins identified with high confidence was 97 ± 70 per sample (341 ± 260 peptides). However, there were two outlier samples (I2 and I5) in which 1538 proteins and 7668 peptides were identified. Conversely, the average number of proteins identified with high confidence in the IVF group was 1640 ± 428 per sample (8543 ± 2613 peptides). This group had a single outlier sample (F3) in which 60 proteins and 163 peptides were identified.

The protein quantification results were visualized using a PCA scores plot (Figure 1C) and a heatmap (Figure 1D). The PCA plot and the heatmap both revealed no clear trends in the composition of the cervical mucus; the first and second principal components of the PCA explained only 15.6% and 10.8% of the variance in the data, respectively. The heatmap had two main clusters, one containing only IUI samples only and one containing samples from both groups. This suggests that the effects of hormone stimulation on the composition of the cervical mucus are outweighed by variation due to other factors.

The Minora feature detector [18] proved to be very useful in the quantitative analysis of the cervical mucus proteomic data. This ProteomeDiscoverer node performs peptide identification based on tandem mass spectra by linking peaks in the spectrum of one sample to the corresponding retention times and parent peptide masses in other samples, thus effectively reducing the number of missing values in the results. When applied to our dataset, it increased the number of quantified proteins in samples with insufficient identifications (i.e., only about 200 proteins identified with a high score). The numbers of proteins identified in the quantitative analysis exceeded those initially identified in the samples, as can be seen in the waterfall graph shown in Figure 1E. In addition, the normalized abundance of proteins in the IUI group was clearly higher than that in both the IVF group and the general proteome. Thus, although the IUI samples contained fewer different proteins than the IVF samples, the proteins that were present were more abundant. The low numbers of proteins in the IUI group could be for instance due to the expression of proteins with variable molecular weight and thus altered diffusion capacity into the cervical mucus of IUI versus IVF samples. To test this hypothesis, we focused on the 63 proteins that were quantified in all replicates of all samples and calculated the correlation between their MW and their measured abundance (Figure 1F). However, this revealed no significant differences between the IUI and IVF groups.

Upon applying common criteria for identifying enriched proteins (a log2 fold change of at least 1 and a *p* value below 0.05), we identified 199 and 422 proteins that were enriched in the IUI (Table S2) and IVF samples, respectively (Table S3). These numbers are higher than the average counts of identified proteins in the IUI samples. We therefore examined the non-imputed datasets of quantified proteins in the IUI- and IVF-enriched protein groups. The finding that fewer proteins were identified in the IUI samples was supported by the results for the IVF-enriched proteins, of which 214 were specific to the IVF group. Another 34 proteins were present only in the IVF group and the positive outlier samples I2 and I5 from the IUI group, while the remaining IVF-enriched proteins were identified in both groups. The intensities of the IUI-enriched proteins were distributed homogenously across the samples.

### 2.4. Protein Annotation of Identified Proteins

**Figure 2:**
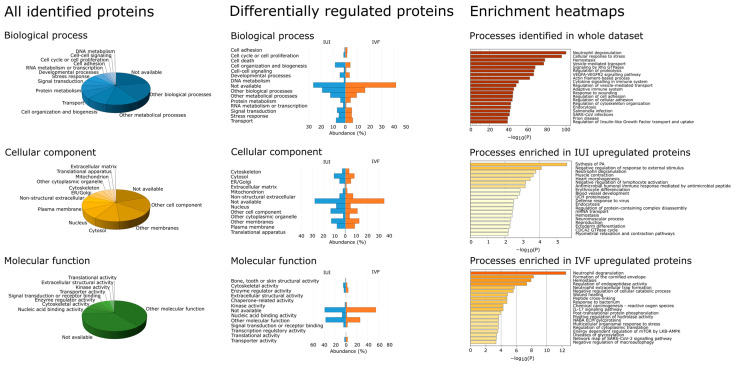
Protein annotation results for the cervical mucus proteome. Annotations for the three main GO term categories—biological function, cellular component, and molecular function—were generated using ProteomeDiscoverer for the complete cervical mucus proteome and for the sets of proteins exhibiting differential expression in the IUI and IVF samples. Additional annotations based on the Metascape [19] enrichment heatmap were also obtained for these three protein sets.

The protein annotation analysis was performed in ProteomeDiscoverer and Metascape (Figure 2). When considering the full set of proteins identified across all samples (representing the general cervical mucus proteome), the most frequent biological process annotations were other biological (18%) and other metabolic (13%) processes, followed by cell organization (8%), transport (7%), protein metabolism (6%) and signal transduction (6%). The most frequent cellular component annotations were other cellular components (15%), other membranes (13%), and cytosol (10%), followed by the nucleus (8%), plasma membrane (8%), non-structural extracellular (6%) and ER/Golgi (5%). The most frequent molecular function annotation was other molecular function (39%), followed by nucleic acid binding activity (6%), cytoskeletal activity (4%), enzyme regulator activity (4%), and signal transduction or receptor binding (4%). No GO annotations were obtained for around 20% of the proteins, which demonstrates a knowledge gap we have in understanding cervical molecular physiology.

The annotation distribution for the set of IUI-enriched proteins was similar to that for the general cervical mucus proteome in all three GO main branches. The biological process annotations exhibiting the most pronounced differences in frequency between the IUI-enriched and general protein sets were no known biological processes (+4.4% in the IUI-enriched set), other biological processes (+0.8%), signal transduction (+0.7%), cell adhesion (−1.4%), other metabolic processes (−1.3%) and protein metabolism (−2.31%). The frequencies of all other biological process annotations in the IUI-enriched set were comparable to those in the general set. The only cellular component annotation whose frequency in the IUI-enriched set differed markedly from that in the general set was no known cellular component (+8.3% in the IUI-enriched set); the frequencies of all other cellular component annotations differed by <2% between the two sets. The only molecular function annotation whose frequency in the IUI-enriched set differed markedly from that in the general set was no known molecular function (+3.2%); the frequencies of all other annotations differed by <2% between the two sets.

Cell process annotations whose frequencies in the IVF-enriched protein set differed substantially from those in the general set were no known biological process (+20% in the IVF-enriched set), cell organization (−4.5%), developmental processes (−1.5%), DNA metabolism (−0.7%), other biological processes (−2%), other metabolic processes (−4.5%), protein metabolism (−2%), RNA metabolism (−1.5%) and transport (−1%). Other cell process annotations (e.g., cell adhesion, cell cycle, cell–cell signaling, signal transduction, and stress response) had similar frequencies in the two sets. Similar results were obtained for cellular component annotations—the annotations exhibiting substantial differences in frequency were no known cellular component (+16% in the IVF-enriched set), cytoskeleton (−1%), cytosol (−2%), ER/Golgi (−1%), mitochondrion (−1%), nucleus (−4%), other cell components (−5%), other membranes (−1%), and translation (−1%). The remaining cell component annotations (plasma membrane, non-structural extracellular, other cytoplasmic organelles, and extracellular matrix) had similar frequencies in the two sets. The molecular function annotations whose frequencies differed markedly between the IVF and general sets were no known molecular function (+20% in the IVF set), cytoskeletal activity (−2.1%), nucleic acid binding activity (−4.2%), other molecular functions (−12.6%), signal transduction activity or receptor binding (−1.2%), and transporter activity (−0.8%). The other molecular function annotations (bone, tooth, or skin structural activity, enzyme regulator activity, extracellular structural activity, kinase activity, and translational activity) had similar frequencies in the two sets.

Because of the high number of “other” process annotations (which do not enable further analysis) in the ProteomeDiscoverer results, we used the Metascape software to acquire additional annotations. This algorithm combines functional enrichment, interactome analysis, gene annotation, and membership search to leverage over 40 independent knowledgebases within a single integrated portal [19] and generates results that can be visualized in the form of enrichment heatmaps (see Figure 2). Annotations assigned to the general cervical mucus proteins in the Metascape analysis included neutrophil degranulation, cellular response to stress, hemostasis, vesicle-mediated transport, and signaling by Rho GTPases.

In accordance with the results for the general cervical mucus proteome, the most enriched annotation in the enrichment heatmap for the differentially expressed proteins in the IVF samples was neutrophil degranulation, and the third most enriched was hemostasis. However, the order of enrichment for the other annotations of the IVF set differed: the second most enriched annotation was formation of a cornified envelope, the fourth was regulation of endopeptidase activity, and the fifth was neutrophil extracellular trap formation. The high number of proteins without annotation can also be explained by the fact that 46% of all differentially regulated IVF proteins were immunoglobulins.

The enrichment heatmap also revealed differences between the annotation distributions of the IUI-enriched proteins and the complete cervical mucus proteome. The annotation exhibiting the greatest enrichment in the IUI set was the synthesis of phosphatidic acid, an essential precursor of phospholipids. The second most enriched annotation was negative regulation of response to external stimulus, followed by neutrophil degranulation processes, muscle contraction, and heart morphogenesis.

Unfortunately, no annotations were obtained for around 20% of the 4370 proteins identified in the full cervical mucus proteome. This was attributed to two factors. First, 1716 of these proteins lacked a gene symbol assigned by the Uniprot database. Second, 1178 of them were identified as immunoglobulins (mostly variable regions). Only 34 Ig proteins have a gene symbol, and to the best of our knowledge, there is currently no good tool for the biological interpretation of different immunoglobulin variants.

### 2.5. Proteins Discriminating IUI and IVF Groups

Separate from the biological interpretation of the proteomic data is the question of which proteins most effectively discriminate IUI from IVF and thus physiological versus hormone-stimulated ovarian cycle. To identify such discriminatory proteins, we used the Wilcoxon exact test and Fisher’s test. This revealed that 29 normalized and imputed source proteins were detected in at least two replicates of all analyzed samples (Table 2), of which 14 were enriched in IUI and 15 in IVF. Four proteins were tissue enhanced in FRT, while some others exhibited group-enriched tissue expression in tissue groups that included the uterine cervix.

**Figure 3:**
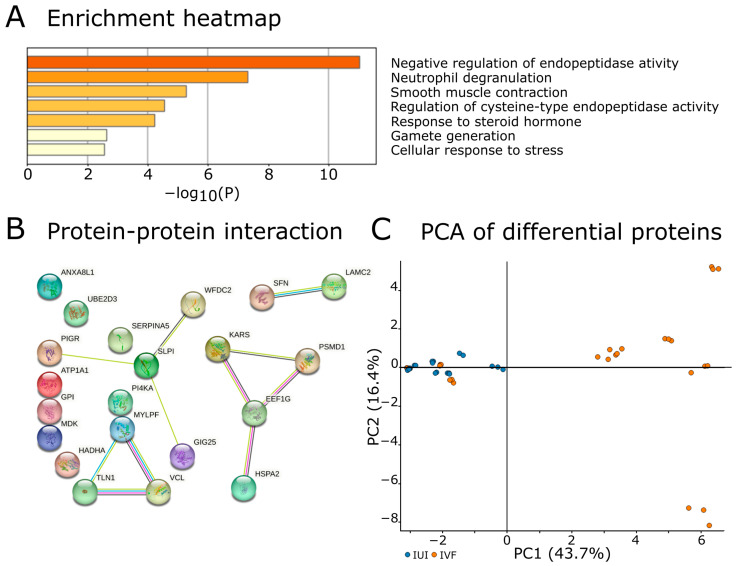
Annotation of proteins with Wilcoxon scores below 0.05. The biological annotations were used to generate an enrichment heatmap with Metascape (**A**) and to map protein–protein interactions (**B**). The PCA plot (**C**) shows the distribution of differentially expressed proteins with Wilcoxon *p* values below 0.05 in the IUI and IVF samples.

Of the 29 proteins listed in Table 2, 23 were recognized by Metascape (Figure 3). Eight were annotated for negative regulation of endopeptidase activity (GO:0010951) with a log_10_ *p* value of −11.03, while seven were annotated for involvement in neutrophil degranulation (R-HSA-6798695; log_10_ *p* value −7.30). No other annotation was assigned to more than four proteins in this group. Four protein–protein interactions were identified among the 29 proteins in an analysis performed using the String platform. The first interaction involved KARS, PSMD1, EEF1G, and HSPA2, all of which are factors influencing protein synthesis and degradation. The second involved WFDC2, SLPI, GIG25 (SERPINA3), and PIGR, which are a group of secreted proteinase inhibitors and an immunoglobulin receptor expressed in mucosal epithelial cells; these proteins were enriched in the IVF group. The third involved MYLPF (MYL11), TLN1 and VCL, which are cytoskeletal proteins involved in cellular adhesion and muscle contraction. The final interaction involved the extracellular matrix proteins SFN and LAMC2.

## 3. Discussion

This study was the first proteomic analysis of human cervical mucus describing differences in composition of cervical mucus sampled during the periovulation period from women undergoing a natural ovarian cycle (IUI group) versus those with synthetic estrogen-induced artificial cycle (IVF group). Although there were earlier efforts in the analysis of cervical mucus reported in 2007 [8,21,22], they identified much less proteins compared to our study (685, 194, and 147 in the studies of Shaw, Anders-Björkmann, and Tang, respectively). Panicker demonstrated an alternative approach in 2009 [9] by using SELDI-TOF to identify 30 protein peaks on average in a mass frame of 2.5–30 kDa. More recently, Grande investigated proteomic changes in the cervical mucus during the menstrual cycle [10] and changes in protein expression associated with endometriosis [11], resulting in the identification of 81 and 140 proteins, respectively. Additionally, Ma searched for protein biomarkers of cervical adenocarcinoma in cervical mucus, identifying 711 proteins [23]. Proteomic analyses of cervical mucus have also been conducted in the context of veterinary medicine [24] and in studies on the cervical plug during pregnancy [25]. The most recent research in the field at the time of manuscript preparation was published by Leo Han, who identified 3048 proteins [26] in a comparative analysis of cervical mucus from humans and rhesus macaques. All of these studies used different approaches to various aspects of sampling and proteomics analysis and thus have different strengths and weaknesses when compared to this work, as we discuss below.

### 3.1. Model System for Cervical Mucus Evaluation

Previous analyses of cervical mucus were performed by collecting samples during spontaneous cycles, which made it impossible to precisely determine the time elapsed between ovulation and sampling; the average error was approximately one day [8,9,10]. The IUI sampling performed in this work had a similar level of accuracy, providing samples reflecting the basal composition of the cervical mucus during normal ovulatory cycles.

Nonetheless, our model system based on IVF/ICSI/ET (the IVF group) mimicked natural conception under standardized conditions but enabled sampling within a window of roughly 15 min around the time of ovulation. This accuracy was possible because the ovaries were monitored by ultrasound, the timing of ovulation was controlled by the application of exogenous hCG, and eggs were collected by ovum pick-up. Cervical mucus samples were acquired immediately prior to egg collection.

### 3.2. Cervical Mucus Sampling

Two main approaches to cervical mucus sampling were used in earlier studies. The first was to collect cervicovaginal fluid or lavage. This approach was used by Shaw [21], who analyzed cervicovaginal fluid obtained by inserting a gauze into the vagina for 1 h, and by Tang [22], who performed vaginal lavage with 5 mL of saline solution. Both approaches are straightforward and provide samples free from compounds that may interfere with subsequent processing. However, the obtained proteome is more of vaginal origin than cervical. The second approach involves extracting cervical mucus by suction with a thin catheter and was used by Andersch-Björkman [8], Grande [10,11], Han [26], and our group. This approach enables selective collection of cervical mucus with minimal contamination from other compartments of the female genitourinary tract. Finally, during regular gynecological examinations conducted [27] to prevent cervical cancer, cervical epithelia are commonly sampled together with fluid/mucus using a specialized brush. This approach was used by Panicker [9], but Andersch-Björkman [8] has stated that it results in relatively severe sample contamination with epithelial and blood cells.

### 3.3. Proteomic Approaches in Cervical Mucus Analysis

The viscosity of the cervical mucus varies with the phase of the menstrual cycle, and thus, samples must be dissolved in an appropriate solvent to facilitate further processing. Several approaches have been used for this purpose. Dissolution is most straightforward for samples of cervicovaginal fluid, which can be extracted from phosphate-buffered saline and cervicovaginal lavage and which can be processed directly. Both materials can then be centrifuged and submitted to downstream sample preparation. However, the processing of cervical mucus obtained by suction is more challenging. A relatively gentle dissolution method was reported by Grande [10,11], who used 0.2% trifluoroacetic acid followed by centrifugation at 9200× *g*. This approach introduces no compounds that could interfere with subsequent protein assays or digestions but that extract only a few of the proteins in the initial sample, as demonstrated by the comparatively small numbers of proteins identified in studies where the procedure was used. The second approach was used by Andersch-Björkman [8], Han [26], and our group. It involves moderately harsh conditions—a 4% SDS buffer was used in our experiments. Andersch-Björkman et al. bypassed the protein concentration assay by directly loading the dissolved mucus into an electrophoretic gel, while Ma [23] et al. used detergent concentrations similar to those used in our work in combination with a chaotropic reagent (7 M Urea, 2 M Thiourea, 4% SDS, 40 mM Tris-HCl, pH 8.5, 1 mM PMSF, 2 mM EDTA). This treatment was followed by acetone precipitation, dissolution of the precipitated proteins in 8 M urea/100 mM tetraethyl-ammonium bromide (TEAB), and iTRAQ metabolic labeling (which circumvented any need to perform a protein concentration assay).

It was difficult to identify a suitable concentration assay for use in this work because of issues with buffer compatibility. Quantitative mass spectrometry is essential for successful biomarker identification, and while many isotopically labeled approaches [28] have been proposed, we consider label-free quantification to be preferable for high-throughput proteomics. We therefore needed to reliably control the amounts of proteins and peptides contained in samples submitted to processing and mass spectrometric analysis. Unfortunately, the composition of the FASP buffer that we used to solubilize mucus samples (4% SDS, 100 mM DTT) makes it incompatible with most colorimetric protein assays; the BCA assay tolerates detergents but is sensitive to reducing agents, while the opposite is true for the Bradford assay. We ultimately achieved acceptable results with the Thermo Scientific Pierce 660 nm protein assay using the ICDR reagent [29]. To evaluate the efficiency of the subsequent digestion step and to tune the number of peptides injected into the LC-MS system, we performed a standard BCA assay before peptide purification.

Because earlier studies on cervical mucus all used different sample preparation methods and mass spectrometric techniques, they also used different digestion and peptide purification strategies. Panicker [9] used SELDI-TOF, which requires a sample preparation strategy differing markedly from those needed in bottom-up proteomic analyses such as those conducted in the other cited studies. Conversely, Tang [22] used two-dimensional electrophoresis, Andersch-Björkman [8] and Shaw [21] used SDS-PAGE fractionation followed by gel spot/band excision and subsequent digestion. Shaw made this approach almost perfect, performing a series of general proteomics experiments and separate specialized experiments examining mucins and their glycosylation. Grande [10,11] and Ma [23] both used a gel-free approach involving direct in-solution digestion. Unfortunately, however, Ma provided only limited details of this process—the explanation regarding how harsh buffers were diluted before trypsin digestion was particularly sparse.

It has been reported that solution phase trypsin digestion is suboptimal in terms of efficiency and the removal of interfering compounds. Consequently, a range of alternative approaches have been developed to enable more effective digestion. One such approach that has achieved considerable popularity is filter-aided sample preparation (FASP), which was developed by Wisniewski [30]. We used this approach successfully in earlier studies [31] and during the initial phase of this work. We subsequently tested FASP optimization using Lys-C endopeptidase and found that the use of this enzyme significantly increased the number of peptides identified in samples, in accordance with the published claims [32]. A similar approach involving enhanced FASP modification [33] was used by Han [26]. While the capabilities of these two methods have not yet been compared directly, the available data suggest that they both perform well.

### 3.4. Cervical Mucus Proteome

As mentioned above, previous studies on cervical mucus proteomics identified 30–3048 proteins in 3–29 patients. Here, we report the identification of 4370 proteins in cervical mucus samples from 19 patients undergoing assisted reproduction with (IVF) or without (IUI) hormonal stimulation. The analysis of the full set of samples, representing both IUI and IVF groups, provided a couple of interesting findings. First, relatively few proteins were identified with high confidence in some samples, mainly from the IUI group. Further examination showed that the number of proteins that were quantified based on precise mass and retention time data exceeded the number identified through analysis of tandem mass spectra. Nevertheless, the number of quantified proteins in the IUI samples was consistently lower than in the IVF samples. Since the protein quantity injected into the mass spectrometer was identical in both cases, there are two possible explanations for this outcome. The first is that the cervical mucus of the women without hormone stimulation contained fewer but more abundantly expressed proteins than that from women receiving hormonal stimulation, which might contain some proteins that were expressed due to treatment with exogenous estrogens. Alternatively, the physicochemical properties of cervical mucus from women receiving hormonal stimulation might differ from that of women not receiving such stimulation due to differences in factors such as the water content or peptidoglycan matrix. Such differences could affect the permeability of the mucus for secreted proteins and hence its preferential enrichment with proteins of lower molecular mass. However, the results presented in Figure 2E,F suggest that the first hypothesis is more plausible. Aside from these differences and the identification of some proteins commonly enriched in IUI and IVF samples, the two sample groups were relatively similar, and the effect of protein size was not reflected in relative abundancy. This is also demonstrated by the heatmap and PCA results shown in Figure 2.

Andersch-Björkmann [8] identified 195 proteins in cervical mucus, 97 of which were also identified in our study. Moreover, 32 of the 148 proteins identified by Tang [22] were detected in our samples. Grande has published two studies on the proteomics of cervical mucus. The first study [10] compared the protein composition of cervical mucus in different phases of the menstrual cycle and identified 109 proteins, of which 26 were also identified in our samples. Grande’s second study focused on discovering endometrial biomarkers and identified 110 proteins that were also identified by us. However, a further 4261 proteins were identified in this work but not in Grande’s study, and 94 proteins were identified by Grande but not by us. Similarly, when comparing our results to those of Han [26], we find that 1040 proteins were common to both datasets, but 3330 were found exclusively in our dataset and 2008 in Han’s. Despite the different sampling procedures, clinical characteristics, instrumentation and protocols used in all these studies, the limited overlap between the sets of identified proteins (which exists even when comparing this work to older studies with few identifications) suggests that the composition of the cervical mucus is highly variable, and further standardization is needed for future diagnostic use. The “minimal” cervical mucus proteome consisting only of proteins identified in this work and all the earlier studies cited in this paragraph contain just nine proteins: A1BG, ALB, ANXA1, APOA1, HP, LCN2, LYZ, PFN1, and S100A9.

Biological annotation of the cervical mucus proteome revealed that 57% of the proteins identified in this work had an intracellular GO annotation (Figure 3). Moreover, 7 of the 20 ontology terms included in the enrichment heatmap for cervical mucus related to intracellular processes. This was interesting because some reports have described the cervical mucus in the ovulatory phase as a cell-free mucous liquid [34]. Our data are not necessarily incompatible with this statement because the presence of intracellular proteins is not direct proof of the presence of cells; it could instead indicate the presence of subcellular particles, exosomes, or just proteins released by cellular and epithelial turnover.

The primary function of the cervical mucus is to present a physical and immunity barrier to the passage of factors from the extra-uterine environment to the upper FRT while remaining semi-permeable to sperm cells during ovulation. The Metascape enrichment analysis presented here is consistent with this function because the enrichment heatmap of the 20 most probable processes associated with the identified proteins contained four immunity-related terms, and neutrophil degranulation (R-HSA-6798695) was found to be the process most likely to be involved in cervical mucus biology. The defensive function of the cervical mucus was also confirmed by the fact that 1178 of the 4370 proteins identified in this work were immunoglobulins and their fragments. This is consistent with previous reports [35,36,37]. The sub-group analysis of fertilization outcomes was not performed due to limited numbers of pregnant patients.

### 3.5. Differences between IUI and IVF

Earlier studies comparing IUI and IVF responses focused mainly on analyzing pregnancy outcomes [38,39,40], evaluating endometrial responses by measuring endometrium thickness [41], or investigating endometrial biology using electron microscopy, histochemical analysis of endometrial biopsies, or non-targeted proteomic analysis of endometrial fluid [42,43,44]. There have also been studies on changes in the cervical mucus (evaluated using the Insler score [2]) induced by hormonal hyperstimulation similar to that received by our IVF group [45]. Moreover, multiple studies have shown that hormonal contraception can cause changes in the properties of the cervical mucus [46,47], again based on the Insler score. However, to the best of our knowledge, only two studies have directly examined hormone-induced changes in the biological composition of the cervical mucus: Andersch-Björkmann [8] and Grande [10] both investigated the protein composition of the cervical mucus during different phases of the natural menstrual cycle.

Andersch-Björkmann [8] identified 195 proteins, 97 of which were also identified in our study. Their main interest lay in the mucins and the changes in their relative abundance over the menstrual cycle that cause the downregulation of mucins during the ovulation period. Their results showed that all mucins were more abundant in cervical mucus from women undergoing IVF, with MUC5AC and MUC16 having Wilcoxon scores below 0.05.

Grande [10] identified 38 proteins that were described as being constitutively expressed in all menstrual cycle phases. Twenty-five of these proteins were also identified in our samples, and three of them had *p* values below 0.05 indicating differential expression; two were enriched in the IVF samples (WFDC2 and SLPI) and one in IUI (IGHG1). Moreover, 20 of the 42 proteins that Grande described as specific to the pre-ovulatory phase were detected in our ovulatory samples. Two proteins from this group (A2GL and DEFB1) were enriched in the IVF samples, and one (EF1A1) was enriched in the IUI group. Only 10 of the 38 proteins that Grande described as being specific to the ovulatory phase were detected in our samples. Two of these ten proteins, LOX12 and RBGP1, were enriched in the IVF group. In the post-ovulatory phase, we identified 7 out of 17 proteins, none of which were enriched in the IVF or IUI groups. These results show that better proteome coverage allowed us to detect proteins previously considered phase-specific in the ovulatory cervical mucus.

Our results also revealed significant differences in the protein content of cervical mucus from the IUI and IVF patient groups: 199 proteins were found to be enriched in IUI and 422 in IVF. According to ProteomeDiscoverer, the main ontology term associated with proteins enriched in the IUI samples and for those enriched in the IVF samples was “not characterized”. More informative results were obtained using Metascape, which indicated that the main ontology terms associated with proteins enriched in the IUI group were phosphatidic acid synthesis, negative regulation of responses to external stimuli, muscle contraction, and heart morphogenesis. The ontology terms associated with proteins enriched in the IVF samples were more similar to the annotations of the general proteome, although some differences were observed. In particular, the IVF group was associated with formation of the cornified envelope, endopeptidase activity, and neutrophil extracellular trap formation. In addition, 27% of the identified proteins were upregulated when comparing the IUI and IVF groups, while 46% were downregulated, suggesting differences in the immune properties of the cervical mucus under IUI and IVF conditions. This is consistent with the fact that the immune response in the FRT is known to be hormonally regulated [48] and our finding that immunoglobulins comprised roughly a quarter of the proteins identified in this work. Useful insights into the effects of IVF and hormonal changes more generally on the immune properties of the cervical mucus could potentially be obtained by comparing our results to those of an earlier study that measured the concentrations of interleukins and cytokines in endometrial fluid from women undergoing IUI and IVF [49]. However, directly comparing the results of these two studies might be difficult because the earlier study also showed that the levels of interleukins and cytokines in endometrial fluid differed substantially from those in cervical mucus. We have not found any other study focused on the description of CM composition after controlled ovarian hyperstimulation. The indirect evidence of how CM changes after hyperstimulation could be found in works from Insler [2], describing the changes of CM during the ovulatory cycle, and in Devroey [50] describing endometrium response to controlled ovarian hyperstimulation.

The method described here could be used in future biomarker discovery studies using CM as a non-invasive biological material. For that purpose, the protocol contains several quality-control steps allowing for data normalization and direct comparison of results: (i) in-depth clinical examination and standardized data collection and mining; (ii) precise timing of CM collection during (peri)ovulatory period; in the IVF, it is the time of ovum pick-up, while in the IUI it is the ultrasound-confirmed ovulation; (iii) standardization and quality assurance process in the laboratory analysis, which includes quantitative measurements of both proteins and peptides. This allows for normalization in the sample preparation and assures that the identical protein quantities are processed and the same number of peptides are subjected to the LC-MS analysis. The quality and accuracy of the LC-MS is assured by weekly calibration and maintenance and is monitored by injection of control BSA samples during the data acquisition. The data processing is performed using the LC-MS vendor’s software for the whole dataset in one batch, using normalization and imputation for subsequent statistical analyses.

The methodology reported here achieved, thus far, the most comprehensive results in proteomic analysis of the cervical mucus. Technically speaking, we have detected a similar or higher number of identified proteins using comparable protocols as in Han’s study [26]. Nonetheless, we have analyzed a much larger cohort of healthy and clinically well-characterized women to establish a reference cervical mucus proteome in the periovulatory period, which will be used in future studies to identify robust disease protein biomarkers.

## 4. Materials and Methods

**Figure 4:**
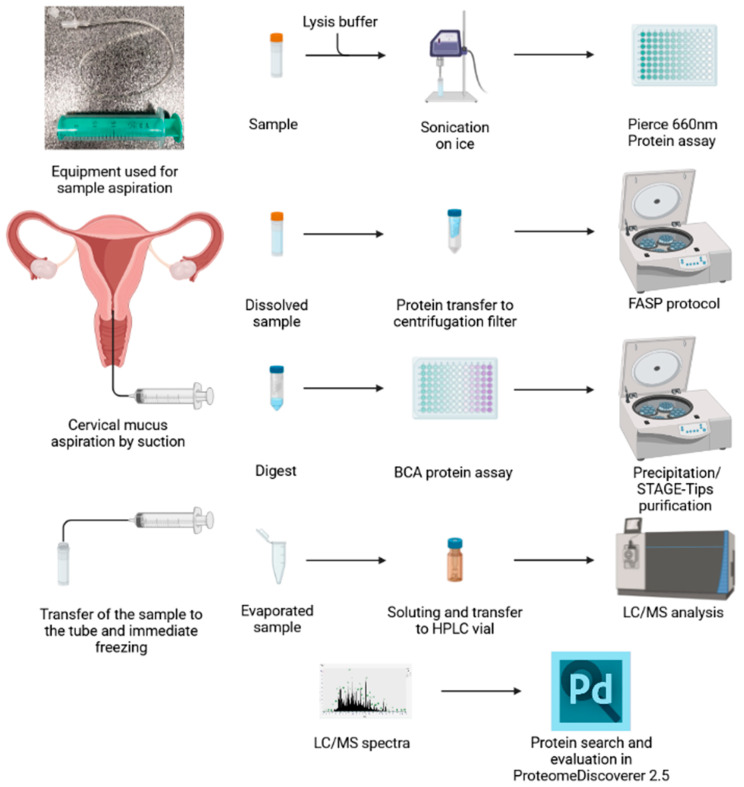
Schematic representation of the cervical mucus processing workflow. Cervical mucus is aspirated by suction using a neonatology umbilical cannula and an injection syringe and then flushed out to the screw tube, which is immediately frozen in liquid nitrogen. After thawing, the mucus is diluted, sonicated, and digested before LC-MS analysis. The figure was created using BioRender.com “www.biorender.com (accessed on 28 November 2022)”.

The workflow for proteomic analysis of cervical mucus is shown in Figure 4.

### 4.1. Patient Criteria

The design of the “Biomarkers of endometrial receptivity (BIOMER, NCT04619524)” trial was approved by the Ethics Committee of the Faculty of Medicine and Dentistry, Palacky University, and University Hospital in Olomouc. Patients at the IVF Unit of University Hospital Olomouc were assessed for eligibility and were invited to enroll in the study. The inclusion criteria were signing informed consent, an infertility diagnosis of male factor or unexplained, and a conception plan based on either (a) the natural ovulation cycle with IUI or (b) a stimulated IVF cycle using recombinant follicle-stimulating hormone (r-FSH), follitropin-alpha (Gonal-F, Merck Europe B.V., Amsterdam, The Netherlands) in combination with a gonadotropin-releasing hormone (*GnRH*) agonist (Diphereline, Ipsen Pharma, Boulogne-Billancourt, France) or antagonist (Cetrotide, Merck Europe B. V., Amsterdam, The Netherlands) as reported elsewhere [50,51].

### 4.2. Sample Aspiration

In the IUI group, patient ovarian cycles were monitored by ultrasound, and IUI was performed during the periovulatory period. Cervical mucus sampling was performed by aspiration immediately before the IUI procedure using a CP-01 neonatal umbilical cannula (Gama Group, catalog no. V646958-ND) connected to a syringe. Samples were then transferred to screw cap tubes (SSIbio, catalog no. 2320-00), immediately frozen in liquid nitrogen and stored at −80 °C until analyzed.

In the IVF group, all patients underwent controlled ovarian hyperstimulation (COH) for IVF/ICSI/ET; 4 patients were stimulated using the GnRH agonist long protocol [51] and 6 with the GnRH antagonist protocol [50]. After the dominant follicle reached 20 mm in diameter, a single injection of 7500 IU of human chorionic gonadotropin (Ovitrelle, Merck Europe B.V., Amsterdam, The Netherlands) was administered, and ovum pick-up was performed 36 h later. Cervical mucus sampling was performed just before ovum pick-up using an identical procedure as in the IUI group.

Patient data were collected in the electronic case report forms (ClinData; “www.clindata.imtm.cz (accessed on 22 November 2022)”.

### 4.3. Sample Dilution

The samples were dissolved in 100 mM Tris-HCl, 100 mM DTT, 4% SDS, pH 7.6 (referred to in the text as FASP buffer) [30] and sonicated using a Sonopuls GM mini 20 (Bandelin, Berlin, Germany) needle sonicator for 1 min using a 1 s pulse/1 s pause sequence with an amplitude of 50% and an energy of 378 J per 1 mL. The protein content of the sonicated samples was then analyzed using the Pierce 660 nm protein assay [29] (Thermo Fisher, Rockford, USA catalog no. 22660) with ionic detergent compatibility reagents (IDCR, Thermo Fisher, catalog no. 22663) in accordance with the manufacturer’s instructions.

### 4.4. Protein Digestion

Samples for proteomic analysis were subjected to filter-aided sample preparation (FASP) as described by Wisniewski [30]. Briefly, each sample was diluted in FASP lysis buffer, transferred to a filter unit (Merck Millipore, Carrigtwohill, Ireland, catalog no. MRCF0R030), mixed with UA buffer (0.1 M Tris-HCl pH 8.5, 8 M Urea), and centrifuged. Unless otherwise indicated, centrifugation was always performed for 15 min at 13,000× *g* and room temperature. The addition of UA buffer and the corresponding centrifugation step were repeated once. After washing, samples were alkylated with 0.05 M iodoacetamide by mixing for 1 min and then left to stand in darkness for 20 min. The samples were then centrifuged, the filtrate was removed, and the samples were washed twice with UA buffer and twice with 0.05 M ammonium bicarbonate (AmBiC). Digestion was performed using Trypsin/Lys-C mix (Promega, Madison, WI, USA, catalog nr. V5073) in 0.05 M AmBiC for 18 h at 37 °C in a water bath. After digestion, the digest was centrifuged for 10 min at 13,000× *g* and washed twice with 0.05 M AmBiC, with each wash being followed by centrifugation at 13,000× *g* for 10 min. Digestion enzymes were used in a 1:100 ratio relative to the protein load. Finally, the peptide concentration of each digest was determined using the BCA protein assay.

### 4.5. Peptide Purification

Ten micrograms of peptides was purified using a two-step protocol. First, 1 mL of ice-cold acetone was added to peptide sample, followed by 20 s vortexing, 10 min of incubation at room temperature, and 10 min of centrifugation at 7000× *g*. After centrifugation, the supernatant was removed, and precipitated peptides were allowed to dry for 1 h. The dry sample was then dissolved in 0.05 M AmBiC and purified using STAGE Tips technology [52] with Styrene Divinyl Benzene reversed-phase sulfonate (SDB-RPS) sorbent. The 200 µL tip was fitted with three layers of Sigma SDB-RPS solid-phase extraction disks (3M, St. Paul, MN, USA, catalog No. 66886-U), and the tip was pressed through the punctured cap of a 2 mL Eppendorf tube. The sorbent in the assembled set was activated with acetonitrile, and the samples were mixed with 1% TFA in water and then with 1% TFA in ethyl acetate. The sample mixture was then loaded into the tip and centrifuged for 3 min at 2000× *g*, after which the flow-through liquid was discarded. The tip with the sample was then washed with 1% TFA in ethyl acetate followed by 0.2% TFA in water, and the flow-through was discarded after each wash. Finally, after being transferred to a new tube, the peptides were eluted using 80% acetonitrile with 1% ammonia. The eluted samples were centrifuged at 2000× *g* for 3 min then vacuum dried and dissolved in 1% acetonitrile with 0.05% trifluoroacetic acid prior to LC/MS analysis.

### 4.6. LC/MS Analysis

One microgram of peptide digest was injected and separated using a Dionex UltiMate 3000 liquid chromatograph (Thermo Scientific, Germering, Germany) and then subjected to MS analysis on a Thermo Orbitrap Exploris 480 instrument (Thermo Fisher Scientific, Bremen, Germany) equipped with the Easy-Spray ion source (Thermo Scientific, Malakka, Malaysia). The HPLC separation protocol consisted of desalting on an Acclaim PrepMap 100 column (100 μm × 2 cm, C18, 5 μm, 100 A; Thermo Scientific, Vilnius, Lithuania) followed by pre-column and analytical separation on a PepMap RSLC column (75 μm × 25 cm, C18, 2 μm, 100 A; Thermo Scientific, Vilnius, Lithuania). Sample loading and desalting was performed using the HPLC’s loading pump at a flow rate of 6 µL/min 1% acetonitrile with 0.05% trifluoroacetic acid. Ten minutes after loading, the column valve was switched, and separation was performed using a nanopump at a flow of 300 nl/min until the 95th minute with a gradient rising from 2% to 35% of mobile phase B. The mobile phases used for separation were 0.1% formic acid in water (A) and 0.1% formic acid in acetonitrile (B). After minute 95, the column was cleaned for 10 min with 95% B and equilibrated with 2% B until minute 125, when the run ended.

The Thermo Orbitrap Exploris 480 was set to use the TopSpeed method with MS acquisition in the orbitrap with a resolution of 120,000 resolution and a mass range of 400–1500. Fragmentation was performed in an orbitrap with a resolution of 15,000 using HCD fragmentation and a 2 s time frame for fragmentation between MS scans. To assure quality control, samples of bovine serum albumin digest were injected and analyzed before and after each batch of experimental samples and at intervals of ten samples during the analysis of the batch.

### 4.7. Protein Search

Raw data processing, including peak list generation and protein searches, was performed using Proteome Discoverer 2.5 (Thermo Fisher Scientific). Peak lists from spectrum files were recalibrated against the Uniprot human database (www.uniprot.org; downloaded on 19 January 2021) with Trypsin (full) digestion and cysteine carbamidomethylation as a static modification. Peak lists were generated using the Spectrum Selector feature with MS1 precursor selection in the 400–5000 Da range and an intensity threshold of 2000 and FTMS as a mass analyzer. The main search was conducted using SequestHT and the UniProt human database (downloaded on 19 January 2021). A maximum of 2 missed cleavages was allowed, and the minimum and maximum peptide lengths were set to 6 and 144 amino acids, respectively. The precursor and fragment mass tolerances were set to 5 ppm and 0.3 Da, respectively. Methionine oxidation, N-terminal acetylation, and cysteine carbamidomethylation were selected as dynamic modifications. The validity of the search was verified using Percolator [53] with the Concatenated target/decoy strategy based on q-values. The maximum delta Cn was set to 0.05 and target FDRs were 0.01 and 0.05 for strict and relaxed criteria, respectively. Chromatographic properties were extracted using Minora Feature detection.

The consensus workflow started with the extraction of identified or quantified peptides from MSF Files. Feature Mapper was used for retention time alignment with a maximum window shift of 10 min and a minimum signal-to-noise ratio of 5. Precursor Ion Quantifier was used to calculate label-free quantification from unique peptides, considering protein groups and using shared quantification results. Precursor quantification was based on ion intensity. Protein abundance was calculated based on the Top 3 Average method. Normalization was set up in the Precursor Ion Quantifier using the Total Peptide Amount method. The second branch of the consensus workflow extracted data from MSF files and focused on peptide and protein validation. The first node in this branch was PSM Grouper with a Site Probability Threshold of 95. The succeeding Peptide Validator used Automatic Validation Mode with a Strict Target FDR of 0.01 and a Relaxed Target FDR of 0.05 for both PSMs and peptides. The validated peptides were processed using Peptide and Protein Filter with Peptide Confidence set to At Least High and a minimum peptide length of 6 amino acids. Keep Lower confidence PSMs and Remove Peptides without Protein Reference were set to False. Protein Filters were set to the minimum number of 2 peptide sequences, counting only rank 1 peptides. The filtered proteins and peptides were then processed in three branches. The first branch led to Protein Scorer, branching to Protein FDR validator (set for Strict FDR 0.01 and relaxed FDR 0.05) and Protein Grouping using the Apply Strict Parsimony function. The second branch led to the Protein Annotation node, set to annotate Biological processes, Cellular Components and Molecular Functions using the same database as the protein search. The final branch led to the Protein Marker node.

All data generated in this work can be obtained via ProteomeXchange using the identifier PXD037654.

### 4.8. Quality Control Metrics

Quality control (QC) analyses of bovine serum albumin digests were processed using Skyline 21.2 (MacCoss Laboratories, Seattle, DC, USA; skyline.ms) [54] using BSA sequences obtained from Uniprot (13. 6. 2022) and reviewed manually. Quality control reports containing the Best Retention time, Total Area MS1, Max Height, Max FWHM (full width–half maximum of peak), and Average Mass Error PPM were then generated for the control samples. The stability of the analytical system was evaluated using the doubly charged peptide YICDNQDTISSK. The criteria for quality acceptance was retention time variability, FWHM and Total Area MS1 relative standard deviation below 15% commonly accepted in LC-MS practice. For the Average Mass Error, we set a limit of 3 ppm internally.

### 4.9. Statistical Analysis

Initial statistical analyses were performed in Proteome Discoverer 2.5 (Thermo Fisher Scientific, Bremen, Germany) and Excel 2016 (Microsoft Corporation, Redmont, DC, USA). The list of identified proteins and peptides was exported to Excel, and identified peptides and proteins were extracted into a single data file containing only peptides identified with high confidence and proteins scored as Master. The counts of peptides and proteins were then averaged over files representing three technical replicates to obtain one value per biological sample. The resulting average values and standard deviations were recorded in a column graph. ProteomeDiscoverer 2.5 was used to perform principal component analysis (PCA) and to generate heatmaps based on normalized protein intensities. The parameters used for heatmap calculation were Scale before clustering, Squared Euclidean distances, and Complete linkage method.

For each sample and protein/peptide, abundance was calculated as the log2 value of the median of the values obtained (from Proteome Discoverer) for three technical replicates or as the median value of the non-missing values (in case of non-imputed abundances). A protein/peptide was considered to be detected in a sample if its abundance was quantified at least in one technical replicate of that sample.

Additional statistical analyses were performed using R, ver. 3.5.2 (Core Team, 2018). The statistical significance of differences in the abundances and detections of each protein/peptide between study groups was evaluated using Wilcoxon’s exact test and Fisher’s exact test, respectively. A *p* value below 0.05 was considered statistically significant.

### 4.10. Bioinformatics Analysis

The bioinformatics analysis consisted of multiple steps. The first step involved determining the biological process, cellular localization, and molecular function of identified proteins. Those protein annotations were performed using ProteomeDiscoverer 2.5 in the Protein annotation node in the Consensus workflow and were visualized using the same program.

Additional annotation of protein functions was performed using the Metascape web interface “metascape.org (accessed on 24 November 2022)” [19]. Uniprot protein IDs exported from ProteomeDiscoverer were uploaded to Metascape together with the Enrichment heatmap and Protein–Protein interaction annotations. The protein-protein interaction was calculated using String web interface [55]. Tissue expression profiles for proteins with Wilcoxon scores below 0.05 were obtained from the Human Protein Atlas database “www.proteinatlas.org (accessed on 9 September 2022)” [20].

## 5. Conclusions

In conclusion, our study defined the cervical mucus proteome thus far in the most comprehensive way. We also validated cervical mucus as a valuable non-invasive source of proximal fluid protein biomarkers and demonstrated the importance of clinical and laboratory standardization processes to enable reproducible measurement and its future use in clinical diagnostics of female reproductive tract disorders.

## Figures and Tables

**Table 1:** Characteristics of the participating patients. Where relevant, values are quoted as means with minima and maxima in parentheses. Age, height, weight and smoker status were obtained during initial visit, AMH and FSH were analyzed from peripheral blood, and endometrial thickness was determined via ultrasound examination at oocyte retrieval procedure. Patients in the IUI group were undergoing intrauterine insemination without hormone stimulation while those in the IVF group were undergoing in vitro fertilization with stimulated cycles. Pregnancy rates were defined as clinically confirmed pregnancy. The population characteristics and hormone levels apply to the date of CM aspiration.

Parameters	All Patients	IUI Group	IVF Group
Count of patients in the group	19	10	9
Age (years)	32.1 (25–43)	33.8 (26–43)	30.2 (25–36)
BMI (kg/m^2^)	25.1 (19–46)	25.7 (19–46)	24.5 (20–33)
AMH (ng/mL)	3.8 (0.8–8.6)	3.5 (0.8–6.8)	4.2 (2.0–8.6)
FSH on the third day (IU/l)	5.7 (2.7–10.9)	6.9 (2.7–10.9)	4.8 (4.1–6.5)
Infertility duration (years)	3.0 (0–7)	3.25 (1–6)	2.6 (0–7)
Number of deliveries	0.4 (0–2)	0.6 (0–2)	0.2 (0–1)
Number of abortions	0	0	0
Smoker (%)	21	30	11
Endometrium on the CM collection day (mm)	9.4 (6–15)	8.6 (6–15)	10.3 (8–14)
Number of retrieved oocytes	12.9 (3–23)/NA ^1^	NA	12.9 (3–23)
Pregnancy rate (%)	26	10	44

^1^ Not applicable in the IUI group. Abbreviations: BMI—body mass index, AMH—anti–Müllerian hormone, FSH—follicle stimulating hormone, CM—cervical mucus.

**Table 2:** The list of proteins distinguishing IUI from IVF cohorts. Those proteins were quantified in at least two replicates of all samples and have Wilcoxon test *p* values below 0.05. The protein intensities in the IUI and IVF columns are reported as medians with the minimum and maximum values of the log2-transformed aggregated intensity shown in parentheses. Log2 fold changes were calculated based on the median values and are reported as IUI/IVF ratios. Tissue expression data are from the Human protein atlas [20].

Protein	Uniprot ID	Gene Symbol	IUI	IVF	Log 2 Fold Change	Wilcox Test *p*-Value	Tissue Expression
WAP four-disulfide core domain protein 2	Q14508	WFDC2	22.23 (21.61–23.18)	28.58 (28.07–30.04)	−6.35	0.00009	Tissue enhanced (cervix, salivary gland)
Enoyl-CoA hydratase	H0YFD6	HADHA	27.4 (26.34–28.32)	22.5 (21.77–24.36)	4.9	0.00015	Tissue enhanced (skeletal muscle)
Dopamine receptor interacting protein 4	Q4W4Y1	DRIP4	24.65 (23.99–29.22)	21.67 (21.64–22.17)	2.98	0.00026	N/A
26S proteasome non-ATPase regulatory subunit 1	Q99460	PSMD1	20.81 (20.02–21.45)	23.23 (22.66–23.39)	−2.42	0.00097	Low tissue specificity
Serpin family A member 3	A0A024R6P0	SERPINA3	23.81 (22.91–24.6)	26.63 (25.33–26.82)	−2.82	0.00097	Group enriched (liver, pancreas)
Connective tissue growth factor	Q5M8T4	CTGF	24.38 (22.83–25.49)	20.38 (20.24–20.76)	4	0.00145	N/A
Glucose-6-phosphate isomerase	P06744	GPI	24.68 (23.75–31.52)	22.16 (21.69–22.44)	2.52	0.00145	Low tissue specificity
Laminin subunit gamma-2	Q13753	LAMC2	25.02 (23.24–25.35)	21.37 (21.04–21.54)	3.65	0.00210	Tissue enhanced (urinary bladder)
Serpin family A member 5	P05154	SERPINA5	23.34 (22.86–24.34)	26.11 (24.62–26.71)	−2.77	0.00299	Tissue enhanced (adrenal gland, liver, testis)
Phosphatidylinositol 4-kinase alpha	P42356	PI4KA	22.81 (21.58–23.46)	20.85 (20.43–21.29)	1.96	0.00299	Low tissue specificity
Antileukoproteinase	P03973	SLPI	29.09 (28.37–30.85)	31.87 (31.66–32.18)	−2.78	0.00567	Group enriched (cervix, salivary gland)
Stratifin	P31947	SFN	22.25 (20.94–23)	24.7 (23.61–25.3)	−2.45	0.00567	Group enriched (esophagus, skin, vagina)
Polymeric immunoglobulin receptor	P01833	PIGR	26.09 (25.25–27.66)	29.5 (28.89–29.86)	−3.41	0.00567	Tissue enhanced (intestine, salivary gland)
Midkine	P21741	MDK	21.56 (21.19–23.06)	24.15 (23.88–24.52)	−2.59	0.00762	Tissue enhanced (ovary)
Complement factor D	Q6FHW3	DF	22.67 (21.52–23.63)	27.12 (23.95–27.79)	−4.45	0.01013	N/A
Heat shock-related 70 kDa protein 2	P54652	HSPA2	22.49 (21.58–23.44)	20.81 (20.43–21.49)	1.68	0.01013	Tissue enhanced (brain, skeletal muscle)
Elongation factor 1-gamma	P26641	EEF1G	25.04 (23.62–25.65)	21.2 (19.37–23.49)	3.84	0.01013	Low tissue specificity
FLJ00385 protein	Q8NF17	FLJ00385	28.94 (26.87–29.81)	26.31 (24.04–26.89)	2.63	0.01013	N/A
Cystatin C	A0A0K0K1J1	CSTS3	24.16 (23.61–25.99)	28.73 (27.02–28.83)	−4.57	0.01327	Tissue enhanced (brain)
Ubiquitin-conjugating enzyme E2D 3	A0A024RDH2	UBE2D3	24.12 (23.36–25.01)	21.1 (19.86–22.89)	3.02	0.01327	Low tissue specificity
Immunoglobulin delta heavy chain	P0DOX3	N/A	23.74 (22.24–27)	21.19 (20.28–21.82)	2.55	0.01327	N/A
Lysine--tRNA ligase	Q15046	KARS1	21.5 (20.19–22.1)	23.81 (21.76–24.82)	−2.31	0.01721	Low tissue specificity
Talin-1	Q9Y490	TLN1	22.05 (21.48–23.09)	23.87 (22.72–24.8)	−1.82	0.02202	Low tissue specificity
Myosin regulatory light chain 11	Q96A32	MYL11	22.61 (21–23.08)	20.21 (19.59–21.23)	2.4	0.02202	Group enriched (skeletal muscle, tongue)
Dermcidin	P81605-2	DCD	24.36 (22.79–27.5)	22.47 (21.57–23.03)	1.89	0.02202	Tissue enriched (skin)
Annexin A8-like protein 1	Q5VT79	ANXA8L1	22.27 (21.4–25.36)	20.01 (19.56–21.26)	2.26	0.02793	Group enriched (esophagus, skin, vagina)
Sodium/potassium-transporting ATPase subunit alpha-1	P05023	ATP1A1	22.94 (22.24–23.32)	21.3 (21.09–22.28)	1.64	0.03499	Tissue enhanced (parathyroid gland)
Vinculin	A0A024QZN4	VCL	22.02 (21.77–22.82)	23.63 (22.4–24.42)	−1.61	0.04347	Low tissue specificity
Keratin 13	A1A4E9	KRT13	24.18 (23.3–24.74)	27.66 (26.01–28.76)	−3.48	0.04347	Tissue enhanced (esophagus, vagina)

## Data Availability

The mass spectrometry proteomics data have been deposited into the ProteomeXchange Consortium via the PRIDE [56] partner repository with the dataset identifiers PXD037654 and 10.6019/PXD037654.

## Supplementary Materials

The following supporting information can be downloaded at: https://www.mdpi.com/article/10.3390/ijms24021038/s1.
